# The HER2 Receptor in Breast Cancer: Pathophysiology, Clinical Use, and New Advances in Therapy

**DOI:** 10.1155/2012/743193

**Published:** 2012-12-20

**Authors:** Zahi Mitri, Tina Constantine, Ruth O'Regan

**Affiliations:** ^1^Department of Internal Medicine, Emory University, Atlanta, GA 30322, USA; ^2^Department of Hematology & Medical Oncology, Emory University, Atlanta, GA 30322, USA; ^3^Winship Cancer Institute, Emory University, Atlanta, GA 30322, USA

## Abstract

Human epidermal growth factor receptor 2 (HER2) is overexpressed in around 20–30% of breast cancer tumors. It is associated with a more aggressive disease, higher recurrence rate, and increased mortality. Trastuzumab is a HER2 receptor blocker that has become the standard of care for the treatment of HER2 positive breast cancer. The effectiveness of Trastuzumab has been well validated in research as well as in clinical practice. The addition of Trastuzumab to standard of care chemotherapy in clinical trials has been shown to improve outcomes for early stage as well as metastatic HER2 positive breast cancer. The most clinically significant side effect of Trastuzumab is the risk of cardiac myocyte injury, leading to the development of congestive heart failure. The emergence of patterns of resistance to Trastuzumab has led to the discovery of new monoclonal antibodies and other targeted agents aimed at overcoming Trastuzumab resistance and improving survival in patients diagnosed with HER2 positive breast cancers.

## 1. Introduction

Human epidermal growth factor receptor HER2 overexpression is present in approximately 20–30% of breast cancer tumors. HER2 overexpression is associated with a more aggressive disease, higher recurrence rate, and shortened survival [1–4]. Trastuzumab is a humanized monoclonal antibody targeting the HER2 receptor, which was approved for use in 1998. The mechanisms of action of Trastuzumab have not been clearly defined, but likely include extracellular mechanisms involving antibody-dependent cellular cytotoxicity (ADCC), and intracellular mechanisms involving apoptosis and cell cycle arrest as well as inhibiting angiogenesis, and preventing DNA repair following chemotherapy-induced damage [5, 6]. Trastuzumab has been shown to be effective in combination with chemotherapy, for the treatment of early stage and metastatic HER2 positive breast cancer.

## 2. Biology

HER2 is part of the epidermal growth factor (EGF) family, along with 3 other receptors: epidermal growth factor receptor (HER1, erbB1), HER2 (erbB2), HER3 (erbB3), and HER4 (erbB4). The HER2 gene is located on the long arm of chromosome 17 and encodes a 185-kDa transmembrane protein [7–9]. The HER2 receptor extracellular domain has no identifiable ligand, unlike the other EGF family receptors. It is present in an active conformation and can undergo ligand-independent dimerization with other EGF receptors [6, 10, 11]. The most active and tumor promoting combination is thought to be the HER2/HER3 dimer [12–15].

The mechanism of action of Trastuzumab is perceived to be through both innate and adaptive immunities. Innate mechanisms lead to cell cycle arrest, with a noted increase in p27 levels, and decrease in cyclin D1 and cyclin-dependent kinase 2 activity [16]. Trastuzumab alone does not seem to promote a significant level of apoptosis, but is synergistic with most chemotherapeutics in preclinical models. This synergism is felt in part to be explained by inhibition of the PI3K/Akt signaling pathway, which normally promotes cell survival [17]. Nevertheless, the innate response alone does not fully explain the effect of Herceptin on tumor regression. Adaptive mechanisms are also present and involve antibody-dependent cell-mediated cytotoxicity (ADCC). This is likely to be T-cell mediated, through activation of the FC receptor, leading to increased cell death [18].

## 3. Clinical Use

Trastuzumab has become the standard of care in the treatment of patient with HER2 positive breast cancer. Data from several randomized trials demonstrated that the addition of Trastuzumab to chemotherapy regimens in the adjuvant setting improves outcome in women with early stage breast cancer [19–22]. In the NSABP B31-NCCTG joint analysis, adjuvant therapy with Trastuzumab for primary operable HER2 positive breast cancer decreases recurrence rates and improves overall survival with a 5-year follow-up analysis [23]. The BCIRG 006 trial showed that the addition of 52 weeks of Trastuzumab therapy to chemotherapy was effective in prolonging disease-free survival and overall survival in patients with early stage HER2 positive breast cancer. This effect was seen with both anthracycline and nonanthracycline-based chemotherapy regimens [24]. In the HERA trial, one year of Trastuzumab following standard chemotherapy was effective in prolonging disease-free and overall survival, at a four-year followup [25]. It is worth noting that patients who crossed over from the observation group to the Trastuzumab arm were also noted to have improved survival. Lastly, the FinHer trial demonstrated that Trastuzumab added to 9-weeks of chemotherapy significantly improved recurrence survival when compared to chemotherapy alone [26].

The ideal timing of Trastuzumab administration appears to be concurrent administration with chemotherapy, based on the NSABP trial [22]. However, this remains controversial since the HERA trial continues to show a benefit for sequential Trastuzumab with a followup of 4 years [25]. In the United States, concurrent chemotherapy and Trastuzumab are most commonly used [27].

Trastuzumab has also been shown to be effective in improving outcome for patients with HER2 positive metastatic breast cancer [28]. This effect has been validated in multiple trials [17, 29]. The Hercules trial [30] showed that combining Trastuzumab with epirubicin and cyclophosphamide resulted in improved response to therapy and prolonged the time to disease progression. The addition of Trastuzumab was also effective in improving survival when used with different chemotherapy regimens, such as docetaxel in the M77001 trial [31]. In the HERTAX trial, the combination of Trastuzumab and docetaxel allowed increased time to disease progression in a higher proportion of patients when compared with single-agent Trastuzumab followed by single-agent docetaxel at disease progression [32]. The HERNATA study again showed that a combination of Trastuzumab with paclitaxel or docetaxel as first line therapy for HER2 positive metastatic breast cancer improved patient overall survival and median time to disease progression [33]. In the BCIRG 007, both regimens (Docetaxel and Trastuzumab v/s Docetaxel, Carboplatin and Trastuzumab) were highly effective in prolonging time to progression of disease, overall survival, and both had acceptable safety profiles [34]. There has also been data that Trastuzumab may be a safe and effective single agent as first-line treatment for HER2 positive metastatic breast cancer [35].

Trastuzumab has been evaluated in combination with hormonal therapy in postmenopausal women with metastatic breast cancer, that is, both hormone receptor and HER2-positive. The TAnDEM study showed that the combination of anastrozole and Trastuzumab was more effective in improving outcomes when compared with anastrozole alone [36].

Trastuzumab has been evaluated extensively in the neoadjuvant setting. A review by Lazaridis et al. presented positive data regarding the integration of Trastuzumab into neoadjuvant treatment regimens [37]. The follow-up results from the GeparQuattro study revealed that higher pathological clinical response was achieved when Trastuzumab was added to neoadjuvant chemotherapy, whether it is an anthracyclines- or taxane-based regimen [38]. These results were validated in other studies, as addition of neoadjuvant Trastuzumab therapy appears to be effective in achieving a higher rate of remission [39–41]. In the NOAH trial, the addition of Trastuzumab to neoadjuvant chemotherapy, followed by 52 weeks of Trastuzumab, was effective in improving event-free survival in patients with HER2-positive locally advanced or inflammatory breast cancer [42].

Lastly, a trial by Buzdar et al. evaluated the effect of adding Trastuzumab to chemotherapy in the neoadjuvant setting for operable HER2 positive breast cancer. The trial was stopped early due to significantly higher rates of pathological response in the Trastuzumab arm [39]. Pathologic complete response appears to be a surrogate marker of outcome for HER2-positive breast cancers, especially those that are ER-negative. There still remain questions to be answered as to the duration, optimal chemotherapy regimen to be used with Trastuzumab therapy, and whether chemotherapy is necessary in combination with Trastuzumab for all patients in the neoadjuvant setting.

## 4. Cardiotoxicity

Cardiac toxicity in patients receiving anthracycline-based chemotherapy has been well established. The mechanism of doxorubicin cardiotoxicity involves direct injury to cardiac myocytes through increased free radicals and oxidative stress [43–45]. Doxorubicin cardiotoxicity is mostly irreversible, which clinically leads to decreased left ventricular ejection fraction (LVEF) and subsequently congestive heart failure symptoms. Due to causing structural damage to the myocytes, doxorubicin therapy renders the myocardium more susceptible to injury and irreversible defects from subsequent stressors, whether their insult is reversible or not [46, 47].

Trastuzumab therapy, unlike anthracyclines, has been associated with reversible injury to the cardiac myocytes. This is mainly due to the fact that it does not appear to cause structural damage to the myocardium [48]. Human epidermal growth factor receptor-2 (HER2/ERB2) is expressed in the adult myocardium and is believed to modulate cardiac function and anthracycline cardiotoxicity [49]. Neuregulin-1 (NRG-1) is a protein essential to cell cycle survival. It triggers a cascade of events that promotes sarcomere stability and relieves oxidative stress [50, 51].

HER2 binds to Neuregulin-1 and initiates this cascade, perpetuating signals to maintain cell survival, response to stress, and prevent apoptosis. Trastuzumab blocks the HER2 receptor, causing these pathways to be interrupted [52–56]. The mechanism of Trastuzumab-induced cardiac toxicity has not clearly been elucidated yet. It is proposed to be a combination of increased oxidative stress as well as inhibiting antiapoptotic mechanisms [57]. The increased oxidative stress seems to occur due to the inhibition of NADPH oxidase, as well as upregulation of the angiotensin II pathways, which in turn cause further inhibition of NRG-1 pathways. All of these events cause an increase in free radicals and subsequently myocyte injury [58, 59]. In addition to that, studies have shown that blockade of the HER2 receptor with Trastuzumab leads to an increased ratio of proapoptotic to antiapoptotic proteins. This leads to shortened cell survival and accelerates apoptosis [60]. These findings, however, do not explain the difference in cardiotoxicity between Trastuzumab and Doxorubicin. Further studies have shown that inhibition of the HER2 receptor leads to changes in the tertiary structure of the cardiac contractile apparatus, but does not induce myocardial cell death. This would explain the transient decline in cardiac function during treatment with Trastuzumab, and recovery with interruption of therapy. This is in contrast to anthracycline toxicity which induces nonreversible changes through maladaptive cardiac remodeling, progressing eventually to heart failure [61–63]. Combined therapy with anthracyclines and Trastuzumab affects a common pathway crucial to cell survival and dealing with oxidative stress. Despite the fact that Trastuzumab-induced myocardial injury is largely reversible, it leaves myocytes more vulnerable to anthracycline-induced toxicity. With the survival pathways inhibited and potentiated by two drugs, cell survival is put at risk, and there is increased risk of apoptosis. This reflects clinically in myocyte death and depressed LVEF, leading to signs and symptoms of CHF [57]. The criteria for Trastuzumab-induced cardiotoxicity was defined in 2002 by The Cardiac Review and Evaluation Committee (CREC). These included (1) cardiomyopathy characterized by a decrease in the left ventricular ejection fraction (LVEF) that is global or more severe in the septum; (2) symptoms of CHF; (3) associated signs of CHF including but not limited to S3 gallop, tachycardia, or both; and (4) a decline in LVEF of 5% to <55% with accompanying signs or symptoms of CHF, or a decline in LVEF of ≥10% to <55% without accompanying signs or symptoms. The presence of any one symptom is enough for the diagnosis of cardiac dysfunction [64]. Trastuzumab has been shown to be associated with improved outcomes in treatment of HER2 positive breast cancer, whether early or metastatic [21]. Several studies have, however, confirmed that there is a higher incidence of cardiac toxicity associated with the use of Trastuzumab. The highest risk seems to occur in the setting of anthracycline therapy followed by Trastuzumab. The combination of a taxane-based regimen followed by Trastuzumab appears to cause less cardiac dysfunction [17, 28, 31, 65]. The myocardial injury associated with Trastuzumab was found to be reversible in patients who were followed for a long period of time after interruption of treatment. Patients were able to recover their LVEF to their pretreatment baseline, and clinical evidence of CHF was resolved [27, 49, 63].

Certain risk factors have been associated with increased risk of cardiac dysfunction with Trastuzumab treatment. These included age >50 years, concomitant use of an anthracycline, hypertension requiring treatment, and post-AC LVEF values of 50–54% [64]. In addition to that, elevation in Troponin levels during the course of anthracycline and Trastuzumab therapy was found to be a strong predictor of subsequent decrease in cardiac function and LVEF. With respect to Trastuzumab therapy, the Troponin rise was seen early on, and affected patients were less likely to regain baseline cardiac function [66, 67]. Given the proven benefit of Trastuzumab therapy with HER2 over expression, research focused on looking for interventions that would help to prevent treatment-induced cardiac toxicity. Researchers first examined nonpharmacological interventions such as aerobic exercise. This was not found to have any benefit in preventing remodeling and cardiac dysfunction associated with Trastuzumab [68–70]. Studies have shown that beta blocker therapy (carvedilol) and angiotensin converting enzyme inhibitors were effective in preventing decrease in LVEF in patients on anthracycline therapy [67, 71]. MANTICORE 101 is an ongoing trial which aims to examine the effect of approved CHF therapy (betablocker, ACEi) on preventing cardiac dysfunction in the setting of Trastuzumab therapy [72].

## 5. Trastuzumab Resistance

Trastuzumab has become widely used for the treatment of HER2 positive breast cancer. Despite the success of Trastuzumab-based therapy in treating all stages of HER2-positive breast cancer, resistance to Trastuzumab is an important issue which affects outcome for a subset of patients. These observations have prompted research into the mechanisms of Trastuzumab resistance, which are thought to underlie failure of therapy [35]. No clear etiology has been identified, but several hypotheses have emerged. First of all, studies have found a truncated HER2 receptor, lacking an extracellular domain, which prevents Trastuzumab binding, hence making it ineffective. Interestingly, the same cell line was successfully inhibited by a combined EGFR/HER2 inhibitor, lapatinib [5, 73, 74].

Lapatinib is a dual EGFR and HER2 receptor tyrosine kinase inhibitor [75, 76]. Lapatinib has been shown to be effective in research in inhibiting growth of breast cancer lines known to be resistant to Trastuzumab [77]. In patients who had documented disease progression on Trastuzumab, the addition of Lapatinib in combination with chemotherapy [78] or with Trastuzumab [79] was effective in prolonging time to disease progression and overall survival. The addition of lapatinib to letrozole for the treatment of hormone positive HER2 positive metastatic breast cancer was superior to letrozole alone in prolonging progression-free survival [80]. Lapatinib is approved in combination with capecitabine for patients with HER2-positive metastatic breast cancer previously treated with Trastuzumab, and in combination with letrozole for metastatic breast cancer that is both hormone receptor and HER2-positive. Pertuzumab is another monoclonal antibody that targets HER2 but attaches to a different site on the receptor and, as such, inhibits heterodimerization between HER2 and other Her receptors [81]. The Cleopatra study randomized patients with metastatic HER2 positive breast cancer to placebo plus Trastuzumab plus docetaxel (control group) or pertuzumab plus Trastuzumab plus docetaxel. The median progression free survival was longer in the pertuzumab group [82, 83]. Pertuzumab is now approved in combination with docetaxel and Trastuzumab for the first-line treatment of HER2-positive metastatic breast cancer. Trastuzumab emtansine (Trastuzumab-DM1) is a new product in which the Trastuzumab moiety binds to HER2 on tumor cell surfaces and, upon internalization, the DM1 moiety is released and binds to tubulin, thereby disrupting microtubule assembly/disassembly dynamics and inhibiting cell division and proliferation. Primary results from the EMILIA study, a randomized trial comparing Trastuzumab DM-1 to lapatinib and capecitabine, have shown that Trastuzumab DM-1 significantly improved progression-free survival in patients with metastatic HER2-positive breast cancer. Further research is looking into inhibiting other targets such as mTOR, P13K, or AKT pathways. Two randomized trials are evaluating the addition of the mTOR inhibitor, everolimus, to chemotherapy and Trastuzumab in the first-line and Trastuzumab-resistance metastatic setting.

## 6. Conclusion

Trastuzumab has been proven to be effective in improving outcomes for patients with early operable as well as metastatic HER2 positive metastatic breast cancer. It has also been shown to increase the pathological response when used in the neoadjuvant setting. Trastuzumab therapy has been associated with an increased risk of cardiac toxicity, especially when used in combination with anthracyclines. Resistance to Trastuzumab therapy has also been documented, as patients have had disease progression while on treatment. A better understanding of the mechanisms of resistance to Trastuzumab therapy constitutes the cornerstone of finding new better tailored therapeutic agents capable of reducing rates of treatment failure and disease progression. Multiple new drugs are under investigation, targeting a wide array of receptors and cell cycle regulatory proteins. Preclinical pharmacological and genomic data will soon make its way into the clinical domain. Integrating this knowledge into therapeutic decision making may change the identity of HER2 positive breast cancer from a one drug for all diseases into a personalized individualized therapy, tailored to each patient's predictable response.
